# Consumption patterns and quantification of folate in commonly consumed green leafy vegetables in Accra, Ghana: a cross-sectional study

**DOI:** 10.1186/s40795-026-01400-6

**Published:** 2026-06-19

**Authors:** Obed Akwaa Harrison, Wu Tianyie, Emma Efua Adimado, Winfred-Peck Dorleku, Firibu Kwesi Saalia, Matilda Steiner-Asiedu, Janet Antwi, Idolo Ifie

**Affiliations:** 1 Department of Nutrition and Food Science, University of Ghana, Legon, Ghana; 2 School of Food Science and Nutrition, University of Leeds, Leeds, UK; 3 Institutional Care Division, Ghana Health Services, Accra, Ghana; 4 Department of Biochemistry, Cell and Molecular Biology, University of Ghana, Legon, Ghana; 5 Department of Food Process Engineering, University of Ghana, Legon, Ghana; 6 Department of Agriculture, Nutrition and Human Ecology, Prairie View A&M University, Texas, USA

**Keywords:** Dietary intake, Dark green leafy vegetables, Folate, High-performance liquid chromatography, Food composition

## Abstract

**Background:**

The B vitamin family of nutrients includes folates; a crucial group of nutrients obtained from food. Folate deficiency is a major concern in Ghana, and the resultant neural tube defects in newborns are rampant, but no surveillance is in place. Dietary approaches have proven more sustainable; therefore, this study aimed to conduct a community survey among women to assess the intake of dark green leafy vegetables (DGLV) and to quantify the folate content in a variety of DGLV frequently consumed in Accra, Ghana.

**Methods:**

A cross-sectional study of 388 pregnant and nursing mothers aged 15 to 49 years in Dodowa, an Accra district, was conducted to analyze their consumption of green leafy vegetables using structured questionnaires. The study also looked at the folate levels of various green leafy vegetables that are widely consumed in Ghana. The Kolmogorov–Smirnov test was used to assess the normality of continuous variables. A variance analysis with a post hoc test was used to see whether there were differences in the means of the readings between samples.

**Results:**

A general low intake of folate-rich vegetables in terms of both frequency per week and quantity consumed was recorded. *Kontomire* emerged as the most frequently consumed DGLV, with 36.1% of participants reporting weekly intake and 24.5% consuming it three times weekly. In contrast, vegetables such as *Gboma, Bokobokor*, and *Alefu* were rarely consumed. Over 87% of participants reported never eating *Gboma*, while 91.5% and 94.3% never consumed *Bokobokor* or *Alefu*, respectively. Folate content in vegetable samples ranged between (44.36 ± 17.37 to 220.39 ± 21.93) ug/100 g and (33.53 ± 9.27 to 122.06 ± 5.19) ug/100 g for fresh and steamed (3 min) samples, respectively. Although the green leafy vegetables studied were found to be rich in folates, steaming led to considerable losses ranging from 8.56 to 78.46%.

**Conclusions:**

The results suggest that raw vegetables may offer higher levels of folate, compared to cooked vegetables. The community survey reported limited consumption of vegetables rich in folate. These findings expand knowledge on neural tube defects, orofacial clefts, and folate content in Ghanaian foods, offering a foundation for future public health measures and policy.

**Supplementary Information:**

The online version contains supplementary material available at https://doi.org/10.1186/s40795-026-01400-6.

## Background

Folate (Vitamin B_9_), a water-soluble vitamin essential for numerous physiological processes, exists in two main forms: naturally occurring folate found in foods and synthetic folic acid, widely used in supplements and fortified foods [1, 2]. Its biological significance extends to DNA synthesis, red blood cell production, and the regulation of homocysteine levels, an essential factor in cardiovascular health [3]. Adequate intake is particularly crucial for women of childbearing age due to its preventive role in neural tube defects (NTDs), congenital malformations of the brain and spinal cord, which typically arise during the first month of embryonic development [4, 5]. NTDs, encompassing conditions such as spina bifida and anencephaly, contribute to over 300,000 affected births annually worldwide, imposing severe health and socioeconomic burdens, especially in resource-constrained settings [2].

The link between maternal folate status and NTD prevention is well-established, leading to fortification programs in over 80 countries to mitigate these defects [6]. However, in many low- and middle-income countries (LMICs) like Ghana, folate deficiency persists as a significant public health issue, compounded by limited laboratory capacity, high costs of nutritional assessments, and inadequate surveillance systems [7, 8]. Local studies indicate NTD prevalence rates of 1.15 to 1.6 per 1,000 live births in regions such as Northern Ghana and Greater Accra [7, 9]. Despite this, there is a paucity of data on the folate content of locally available folate-rich dark green leafy vegetables commonly consumed in Ghana, and little attention has been given to dietary strategies to improve folate intake, even though they offer a cost-effective and sustainable approach compared to fortification and supplementation initiatives [10].

Green leafy vegetables are commonly consumed in Ghanaian households and are acknowledged as rich sources of natural folate. Nevertheless, there remains limited documentation regarding which specific varieties are most frequently consumed [11, 12]. Cooking methods such as steaming and boiling, commonly practised locally, often result in significant nutrient loss due to folate’s water-soluble nature [13]. High-performance liquid chromatography (HPLC) techniques, known for their sensitivity and precision, have become a preferred method for quantifying folate content in food matrices, enabling an understanding of how food processing impacts folate availability [13, 14]. However, Colecraft et al. [15] have reported vegetables to be one of the least consumed food groups, while economic, physiological, and organoleptic barriers, including bitter after taste, nausea, and vomiting, among others, impede the intake of folate-rich foods and supplements [16].

This study surveyed the consumption of dark green leafy vegetables among women of reproductive age in a peri-urban community and further assessed the folate content of commonly consumed green leafy vegetables in Accra, Ghana, under fresh and steamed conditions, to determine their potential contribution to meeting the recommended daily allowance (RDA) for various population groups. The findings provide insights into optimizing dietary practices and cooking methods to preserve folate content, ultimately informing public health strategies to reduce the burden of folate deficiency-related disorders, including NTDs and orofacial clefts (OFCs).

## Materials and methods

### Aim

The main aim of this study was to assess the consumption of dark green leafy vegetables among women of childbearing age in Ghana and also to determine the folate content of these vegetables, either in their raw state or cooked state.

### Study design and setting

The study design comprised both a cross-sectional survey to assess the consumption of dark green leafy vegetables and a laboratory experimentation to quantify the folate content in these commonly consumed folate-rich vegetables.

### Community survey

A descriptive cross-sectional survey was carried out at Dodowa in the Shai-Osudoku District during this part of the study. The Greater Accra Region of Ghana is composed of 10 districts, one of which is the Shai-Osudoku District.

### Study population, sample size, and sampling

Pregnant and nursing women between the reproductive ages of 15 and 49 years made up the study population. The following formula, reported by Charan and Biswas, was used to determine the sample size [17]. The sample size was determined using a 53.8% prevalence of folic acid insufficiency among women in the Ghana Micronutrient Survey [18].

$$\mathrm{N}\;=\;\frac{\mathrm{Z}^{2}\;\times \mathrm{P}(1-\mathrm{P})}{\mathrm{D}^{2}}$$

Where: 

N = the minimum required sample size

Z = the critical value for the 95% confidence level (1.96)

P = the estimated prevalence of folic acid deficiency in Ghana (0.538)

D = the margin of error (0.05)

$$\mathrm{N}=\frac{1.96^2 \times0.538(1-0.538)}{0.05^2}$$

$$\mathrm{N}=381.94 \approx 382$$

To account for the possibility of incomplete or non-response questionnaires, five percent was added to the estimated sample size. This led to an estimate of 400 pregnant and lactating women in the study's total sample size.

### Eligibility criteria

The following inclusion and exclusion criteria were put in place to ensure that the sample population was relevant to the research questions and to improve the validity of the study findings. Participants were included in the study if they were pregnant and lactating women between the ages of 15 to 49 years. Also, participants were permanent residents of the study location and were willing to participate in the survey. Participants were excluded from the study if they were unwell or had recently come to visit the Shai-Osudoku district and had no plans of staying permanently.

### Sampling method

During the study period, 388 pregnant and nursing women of reproductive age in Dodowa were chosen using a modified transient walk and a simple random approach. This approach is similar to the snowballing approach, where the first participant leads to the next eligible participants. Dodowa was purposively selected from the Shai-Osu Doku district because of its peri-urban nature and population that could contribute to a diverse and representative sample size. Dodowa also had a varied distribution of tribes from different parts of Ghana as settlers. The selection of study participants was based on inclusion and exclusion criteria.

### Survey instrument, pretesting, and interviewing

To conduct the survey, a semi-structured questionnaire was employed [see Additional file 1]. Despite being created in English, the questionnaire was administered in both the local dialect and English (Twi, Fante, Ga, or Ewe). This made it possible for non-English speaking pregnant and nursing women to understand the questionnaire and comfortably and appropriately answer it. The neighboring community of Sota served as the pretest location for the questionnaire. The characteristics of the pregnant and nursing women in this community were comparable to those of the women in Dodowa. The questionnaire was pretested on 50 participants, and appropriate modifications were made to the questionnaire considering the replies received to guarantee accuracy, repeatability, and clarity.

The survey was divided into four sections: (1) Background data; (2) Knowledge and awareness; (3) Supplementation with folic acid, and (4) consumption of folate-rich foods. During the interviews, open-ended questions were posed in an impartial and exploratory manner, aiming to gather relevant information on the participants' nutritional status, particularly regarding dietary recalls and estimations. The use of food models and pictures of portion sizes further facilitated the accurate estimation of dietary information.

The brief food frequency questionnaire (FFQ) was administered as part of assessing folate awareness and use was a semi-quantitative questionnaire with the following consumption frequencies: never, once a week, two times per week, three times per week, four times per week, five times per week, six times per week and every day. The prior week was utilized as the reference period. The West African food composition [19] table that details the folate-rich foods in Ghana served as the basis for the food items list. There were food photos included in the FFQ. There were small, medium, and large portion sizes displayed in each shot. Portion size estimation was facilitated using a set of images as opposed to a single photograph or none, which has been linked to lower errors in portion size perception [20, 21]. Participants were asked to select the image that most closely reflected their usual portion size consumed per eating occasion. The selected portion sizes were subsequently converted to gram weights (g) using standardized portion-to-weight conversion factors derived from typical serving sizes of the respective vegetables. Final portion sizes were expressed in grams and used for quantitative dietary intake analysis. The FFQ items and portion size estimation approach were adapted from validated questionnaires previously used in similar dietary studies [20, 21].

### Data collection

Trained field assistants conducted in-person interviews using pretested questionnaires that were uploaded onto Kobo Collect version 2021.2.4 for Android to gather the data. Kobo Collect is an application based on the open-source ODK Collect app, which is commonly used for primary data collection in challenging field environments and humanitarian emergencies. This app enabled the storage of data collected from interviews and other primary sources, both online and offline. It offered unlimited capacity for forms, questions, and submissions, including the inclusion of photos and other media, making it a suitable choice for this study. 9

### Data management

To maintain the security and confidentiality of the data collected, appropriate measures were implemented. Hard copies of consent forms were securely stored in locked cabinets with limited access, and personal identifiers were removed from datasets after data analyses. Microsoft Excel was used to generate random numbers and codes as identifiers for each household. The Kobo toolbox suite version 1.30.1, a password-protected platform, was utilized for data management in the field. Coded data were categorized and entered different tables, with numerical subject identifiers assigned to ensure anonymity. Data management steps were recorded in a logbook. The data were saved in Dropbox, a cloud storage platform with restricted access, and a portable hard drive accessible only to the principal investigator and supervisors. Personal identifiers were removed from the files, and all data files were password-protected.

### Quantification of folate content

#### Sample collection and preparation

Samples of nine commonly used green leafy vegetables, namely: Lettuce, African eggplant leaves, Jute leaves, Amaranth leaves, Cocoyam leaves, African spinach, Cassava leaves, Waterleaf, and Dandelion leaves, were collected for folate content determination. Seven of the vegetables are cultivated while two grow wild (Table 1). The selection of these vegetables was based on a previous study by Akwaa Harrison et al. [22] and another unpublished survey that sought to estimate the proximate composition, iron content, and bioavailability of green leafy vegetables commonly consumed in the Accra Metropolis. Vegetable samples were obtained in duplicates between May and June 2022 from 3 different farming sites in the Accra Metropolis district of Ghana. The farm sites were selected based on a previous study by Quansah and colleagues [23], and the areas selected were Haatso (3 farms included), East Legon enclave (3 farms included), and the Roman Ridge residential area (3 farms included). Farmers who produced the vegetables and agreed to take part in the study provided the samples. Eight distinct vegetable samples, each weighing 100 g, were harvested and placed in sterile zip-lock bags (Nasco, Fort Atkinson, WI). They were then transported to the food processing laboratory in the Department of Nutrition and Food Science at the University of Ghana and stored in a cooler (Rubbermaid; Newell Brands Inc, Atlanta, GA, USA) with ice packs (VWR, Lutterworth, UK). Fresh samples were prepared by separating the edible portions and cutting them into smaller sizes. Samples were appropriately labelled with names, farming sites, and dates.

**Table 1:** Selected commonly consumed green leafy vegetables

Number	Common/Local name	Scientific name
1	*Kontomire* (Cocoyam leaves)^#^	*Xanthosoma sagittifolium* (L.) Schott
2	*Ayoyo* (Jute leaves)^#^	*Corchorus olitorius* L
3	*Gboma* (African eggplant leaves)^#^	*Solanum macrocarpon* L
4	*Alefu* (*Amaranth leaves)*^#^	*Amaranthus hybridus* L
5	African Spinach^#^	*Spinacia oleracea* L
6	Lettuce^#^	*Lactuca sativa* L
7	*Bokobokor* (Water Leaf)*	*Talinum fruticosum* (L.) Juss
8	Dandelion*	*Taraxacum officinale* F.H.Wigg
9	*Bankye ahaban* (Cassava leaves)^#^	*Manihot esculenta* Crantz

^#^Cultivated vegetables/ leaves

^*^Traditionally uncultivated vegetables

Fifty grams of edible portions of the vegetable samples were steamed in boiling water using a suspended steamer for 3 min. The samples were processed immediately upon arrival at the laboratory for moisture analysis, and the remaining samples were used for freeze-drying using standard Association of Analytical Chemists (AOAC) methods as described by Ahn and colleagues [24]. They were then homogenized and stored at -80 °C until they were sent for further analysis at the Food Analysis Laboratory, School of Food Science and Nutrition, University of Leeds, England.

### Moisture content determination

The moisture content of the freshly harvested samples was determined by drying to constant weight in a hot air oven (Memmert UN110, Memmert GmbH + Co. KG, Germany) at 105 °C for 24 h, according to the AOAC Official Method 930.15. About 3 g of samples were weighed into previously dried and weighed cans and dried at 70 ˚C until a constant weight was achieved within 72 h. Moisture content was estimated by the difference in weights before and after drying. Thus, dry matter was calculated by subtracting moisture content from 100 (g/100 g).

## Quantification of folates in vegetable samples

### Preparation of samples and stock solutions

The folate content of the selected vegetables was determined as described by Mahato and colleagues and Nojavan and colleagues [25, 26] with slight modifications. The method employed quantifies folate without enzymatic pre-digestion, as extraction conditions were sufficient to release folates from the food matrix. Initially, a stock solution was prepared by combining 20 mg of folate with 200 μL of 1M sodium hydroxide in a 15 mL tube. The addition of sodium hydroxide was to create a pH that would ensure the stability of folate. Next, 10 mL of distilled water was added, and the pH of the solution was adjusted to 6.0 by adding approximately 150 μL of 1M HCl. The mixture was then vortexed for an additional 30s and transferred to a 100 mL amber volumetric flask along with 10 mL of distilled water and 20 mL of ethanol. The final volume was adjusted to the 100 mL mark with distilled water, resulting in a concentration of 200 mg/L. Calibration standards of various concentrations (800, 400, 200, 100, and 50 μg/L) were prepared through serial dilutions from the stock solutions. The dilutions were carried out using a solution composed of 15% acetonitrile in water, along with 0.1% trifluoroacetic acid (TFA). Folates were shielded from oxidation during sample pre-treatment by analyzing in low-light conditions (the laboratory had LED-fitted light bulbs that did not emit UV rays that could destroy the folates) and chilling the sample in ice after every heating.

### Chemical transformation of samples

Two grams of milled vegetable powder was accurately weighed and dissolved in a 25 mL solution mixture containing phosphate buffer (0.1molL^−1^), sodium ascorbate (2%w/v), and.

2-mercaptoethanol (0.1%w/v) adjusted to pH 6.8. The addition of this mixture was to create a pH that promotes the stability of the folates and to prevent oxidation. After shaking, the closed container was centrifuged at 4000 rpm for 5 min to ensure homogeneity. The aqueous phase was separated and filtered, and 1 mL of sample solution was then placed in a 15 mL test tube, and 9 mL distilled water (containing aliquot 0.01%w/v) was added to the sample solution. The pH was adjusted to 11, and a solution consisting of 60 mL 1-octanol (extraction solvent) and 450 mL ethanol (disperser solvent) was injected rapidly by syringe into the extraction device. To separate the organic phase, the liquid mixture was centrifuged for 5 min at 4000 rpm. After this process, the dispersed fine droplets of 1-octanol floated on the aqueous sample. The lower-aqueous phase was separated, and 20 mL of the floated phase was injected directly into the HPLC using a micro syringe.

### Folate quantification

**Fig. 1:**
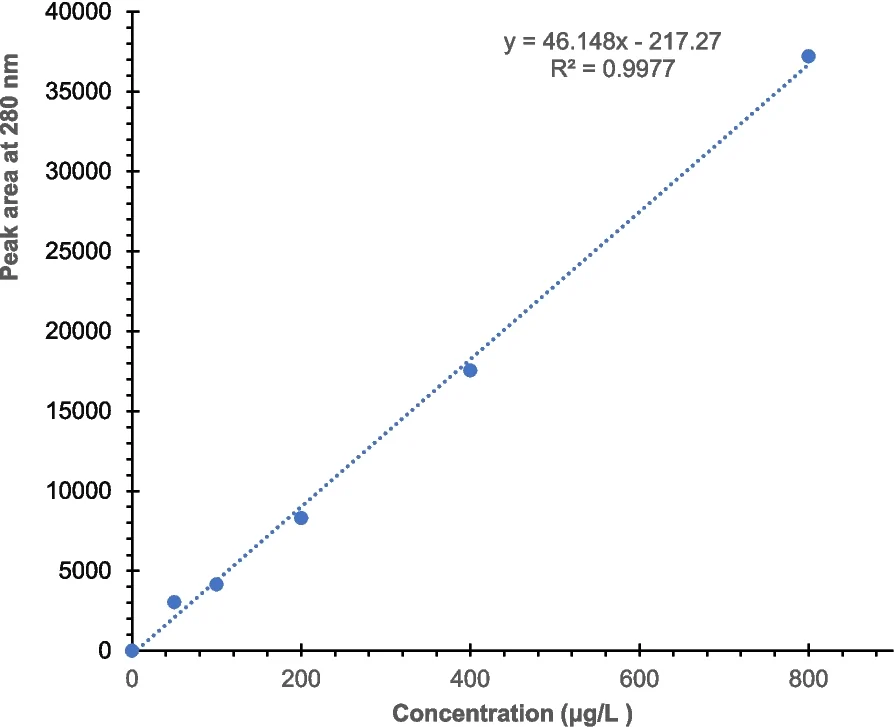
Calibration curve of folate with 800, 400, 200, 100, and 50 μg/L

HPLC identification and quantitation of folates were carried out using a UFLC_XR_ system (Shimadzu) consisting of a binary pump, a photodiode array with multiple wavelengths (SPD-20A), a solvent delivery module (LC-20AD) coupled with an online unit degasser (DGU-20A3/A5), and a thermostat autosampler/injector unit (SIL-20A). A gradient program of mobile phase A (0.1% TFA in water) and organic modifier (acetonitrile) at a flow rate of 0.6 mL/min was used. An initial 7% organic phase was raised to 20% in 5 min with a 1-min hold. Then, it was raised to 65% in 1 min with a hold time of 2 min for the column wash. The organic content was brought down to the initial 7% in the next 1 min, and a hold of 2 min was allowed for equilibration. The photodiode array detector was set to measure at wavelengths (λ-max) 280 nm. The chromatographic separation was performed on a Phenomenex Gemini C_18_ column (5 μm, 250 mm × 4.6 mm) at a flow rate of 0.6 mL/min. The temperature of the column was maintained at 35°C, and the injection volume was 20 μL. Identification of folate in vegetable samples was done based on a comparison with standard folate compounds run under similar conditions in terms of retention time. The linear response range and regression coefficient (*R*^2^) were determined by analyzing the calibration standards (Fig. 1) and fitting the data into a linear regression curve, including zero as the reagent blank.

### Data analyses

The Statistical Package for Social Sciences (SPSS) software version 27 for Windows was used for analysis. Normality was tested for continuous variables using the Kolmogorov–Smirnov test. Parametric data variables were summarized into mean ± SD, whereas.

non-parametric data were reported as median and interquartile range. Analysis of variance at a 95% significance level, followed by a post hoc test, was used to determine differences between sample means.

## Results

### Consumption of folate-rich vegetables among participants

The survey assessed the frequency and quantity of dark green leafy vegetable (DGLV) consumption among 388 participants in a peri-urban community, revealing notable disparities in dietary patterns. *Kontomire* emerged as the most frequently consumed DGLV, with 36.1% of participants reporting weekly intake and 24.5% consuming it three times weekly (Table 2). However, daily consumption was strikingly low (1.0%), and 9.0% never consumed it. Similarly, *Ayoyo* was consumed weekly by 51.0% of participants, though daily intake remained minimal (1.3%). Median quantities for both *Kontomire* and *Ayoyo* were 40 g per serving, with wide variability in individual consumption (ranges: 0–466 g and 0–463 g, respectively).

**Table 2:** Frequency of consumption of folate-rich vegetables among participants (*N* = 388)

Variable	Never n (%)	1/week n (%)	2/week n (%)	3/week n (%)	4/week n (%)	5/week n (%)	6/week n (%)	Everyday n (%)	Quantity (g) Median (Range)
Dark GLV									
* Kontomire*	35 (9.0)	140 (36.1)	87 (22.1)	95 (24.5)	13 (3.4)	14 (3.6)	0 (0.0)	4 (1.0)	40 (0–466)
* Ayoyo*	134 (34.5)	198 (51.0)	19 (4.9)	24 (6.2)	0 (0.0)	3 (0.8)	5 (1.3)	5 (1.3)	40 (0–463)
* Gboma*	399 (87.4)	33 (8.5)	5 (1.3)	8 (2.1)	3 (0.8)	0 (0.0)	0 (0.0)	0 (0.0)	18 (0–461)
Bokoborkor	355 (91.5)	16 (4.1)	10 (2.6)	0 (0.0)	0 (0.0)	0 (0.0)	4 (1.0)	3 (0.8)	20 (0–138)
* Alefu*	366 (94.3)	5 (1.3)	9 (2.3)	0 (0.0)	0 (0.0)	3 (0.8)	5 (1.3)	0 (0.0)	17 (0–462)
Lettuce	213 (54.9)	156 (40.2)	13 (3.4)	3 (0.8)	3 (0.8)	0 (0.0)	0 (0.0)	0 (0.0)	33 (0–468)
Spring onion	130 (33.5)	33 (8.5)	68 (17.5)	72 (16.5)	13 (3.4)	8 (2.1)	5 (1.3)	59 (15.2)	24 (0–378)
Spinach	269 (69.3)	66 (17.0)	14 (3.6)	3 (10.1)	0 (0.0)	0 (0.0)	0 (0.0)	0 (0.0)	14 (0–466)
Dandelion	282 (72.7)	85 (21.9)	15 (3.9)	3 (0.8)	0 (0.0)	0 (0.0)	0 (0.0)	3 (0.8)	25 (0–466)

*GLV* Green Leafy Vegetables

In contrast, vegetables such as *Gboma*, *Bokobokor*, and *Alefu* were rarely consumed. Over 87% of participants reported never eating *Gboma*, while 91.5% and 94.3% never consumed *Bokobokor* or *Alefu*, respectively. Spinach, another folate-rich vegetable, was never consumed by 69.3% of participants, and only 10.1% ate it three times weekly. Dandelion greens followed a similar trend, with 72.7% never consuming them. Spring onions stood out as an exception, with 15.2% of participants consuming them daily and 33.5% reporting weekly intake.

Median consumption quantities varied significantly across vegetables, ranging from 14 g for spinach to 33 g for lettuce. Notably, consumption ranges spanned from 0 g (indicating non-consumers) to over 460 g for certain vegetables, reflecting extreme heterogeneity in dietary habits. These findings underscore a heavy reliance on a limited selection of DGLVs, such as *Kontomire* and *Ayoyo*, while nutrient-dense options like spinach and *Alefu* remain underutilized.

### Evaluation of folate content in commonly consumed green leafy vegetables

The folate content of nine commonly consumed green leafy vegetables was assessed. These vegetables were evaluated for their folate content in both raw and steamed forms. Before folate content estimation, moisture content was estimated to account for variations in folate content calculations due to the differences in the moisture content of the vegetables and the water-soluble nature of folate. Samples were freeze-dried to a stable moisture of 4% to ensure that the analysis was done on a dry matter basis, which ensures a more accurate and uniform folate content estimation. Freeze-drying was also done for ease of storage and transport. 

### The moisture content of vegetables

**Fig. 2:**
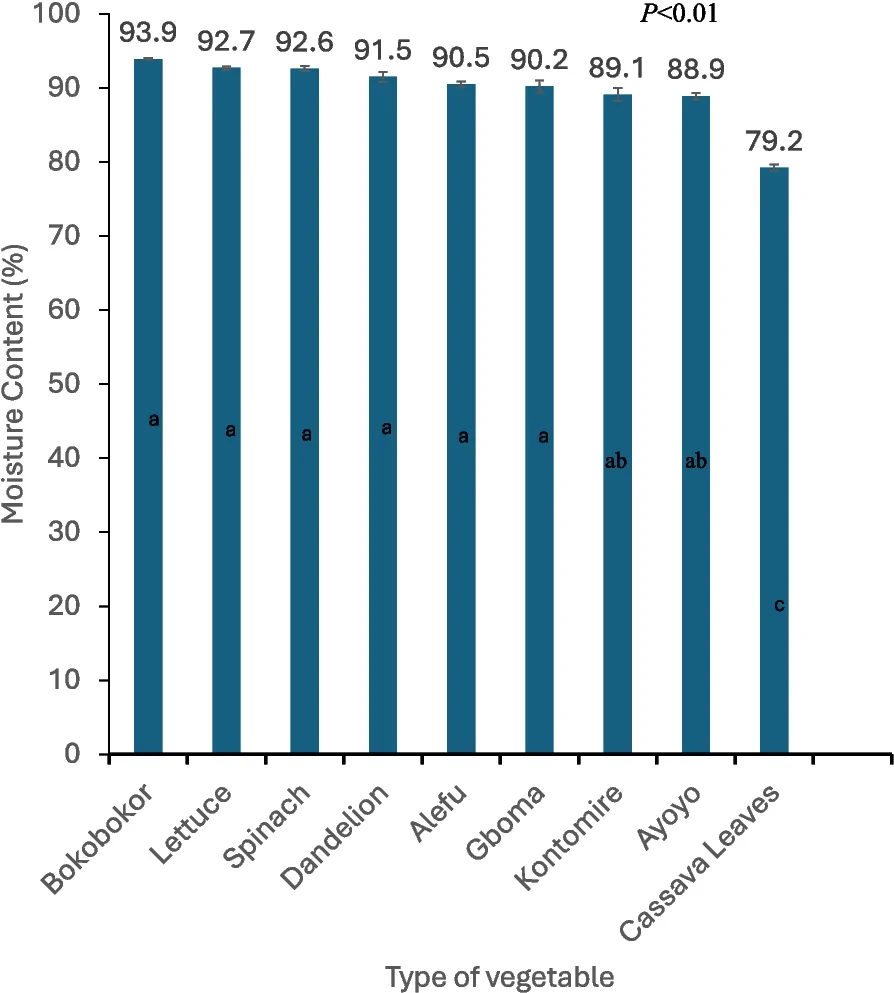
Moisture content by vegetable type

Figure 2 illustrates the moisture content of selected vegetables on an “as is bases”. The data show that *Bokobokor* had the highest moisture content at 93.92 ± 0.39%, while Cassava Leaves had the lowest moisture content at 79.20 ± 0.68%.

### The folate content of selected fresh and steamed vegetables

**Fig. 3:**
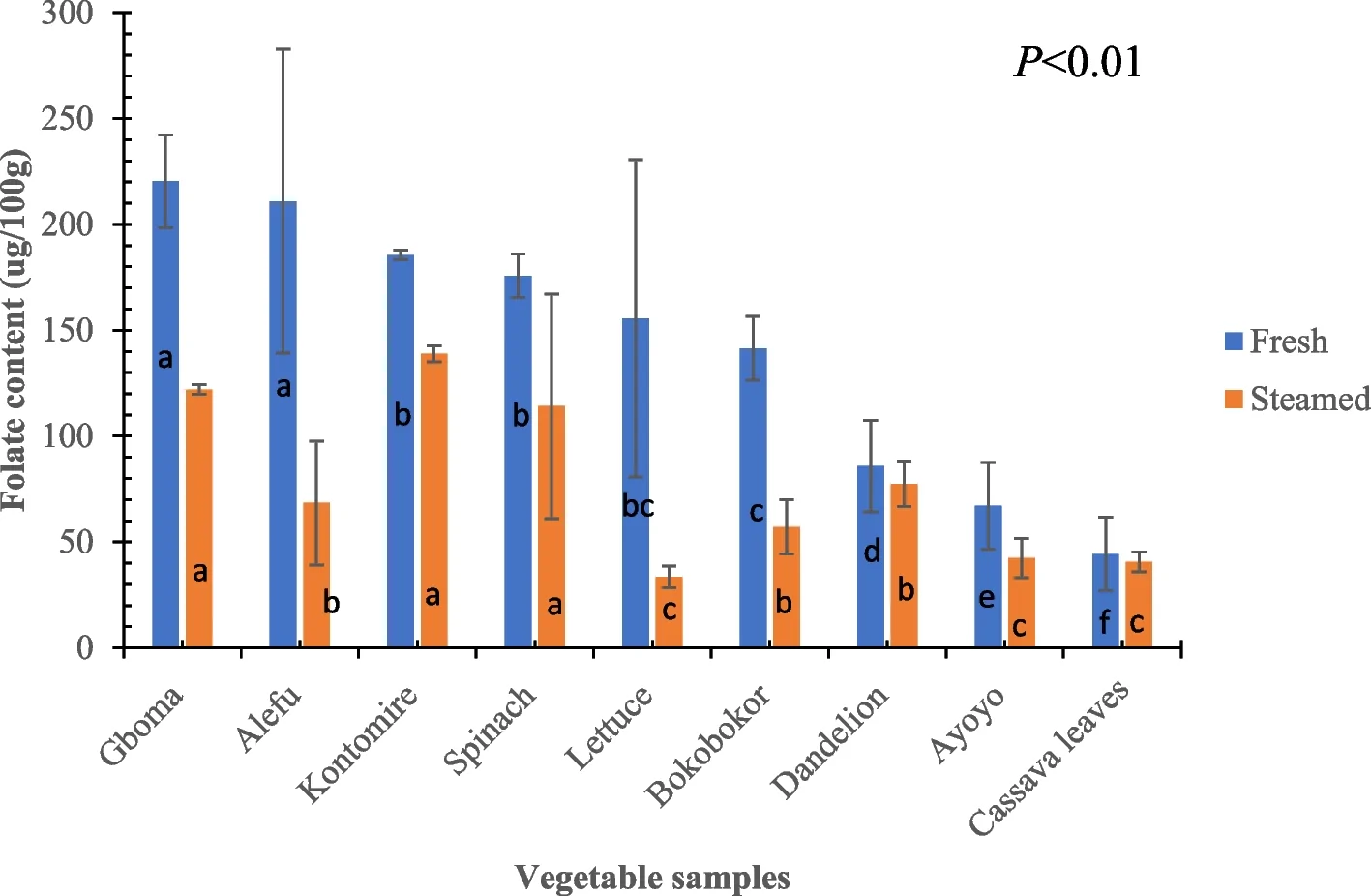
Folate content by vegetable type and treatment

**Fig. 4:**
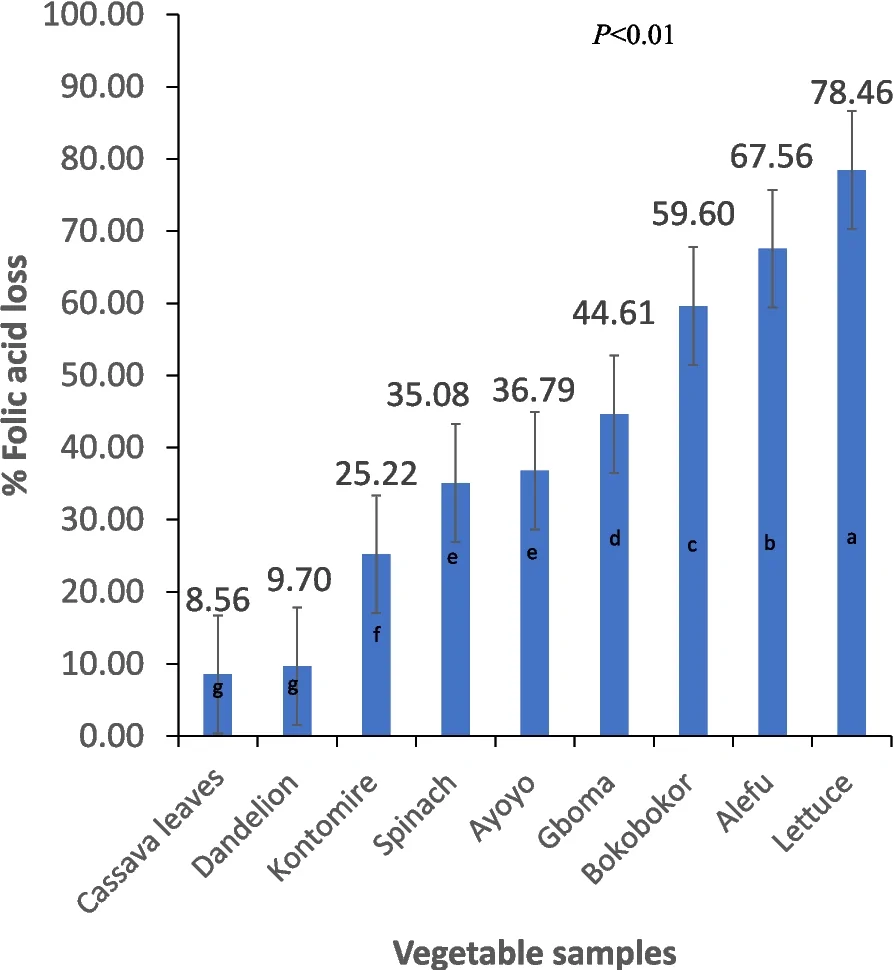
Folate losses by vegetable type after steaming

The results in Figs. 3 and 4 show that *Gboma*, *Alefu*, *Kontomire* and Spinach, had the highest folate content ((220.39 ± 21.93, 210.84 ± 71.73, 185.55 ± 2.27 and 175.71 ± 114.07) ug/100 g respectively) whilst the least was found in cassava leaves (44.36 ± 40.57 ug/100 g). Steamed samples generally had lower folate content.

(Fig. 3) and higher folate loss compared to fresh samples (Fig. 4). Lettuce (78.46%), *Alefu* (67.56%), and *Bokobokor* (59.60%) had the highest folate losses when steamed, whilst cassava leaves and dandelion recorded the lowest losses (8.56% and 9.70% respectively). The folate content of the raw vegetables could provide up to nearly (11 to 55) %, (7 to 36) %, and (9 to 44) % of the RDA for non-pregnant, pregnant, and lactating women, respectively, when 100 g is consumed (Table 3).

**Table 3:** Folate content and losses of fresh and steamed vegetables (ug/100 g)

**Fresh samples**					**Steamed samples**				
**Vegetables**	**Mean ± SD**	**% RDA**^**a**^	**% RDA **^**b**^	**% RDA **^**c**^	**Mean ± SD**	**% RDA**^**a**^	**% RDA**^**b**^	**% RDA**^**c**^	**% B**_**9**_** Loss**^**d**^
*Spinach*	175.71** ± **10.34	43.93	29.29	35.14	114.07** ± **2.24	28.52	19.01	22.81	35.08
*Dandelion*	85.83** ± **21.52	21.46	14.30	17.17	77.50** ± **29.18	19.38	12.92	15.50	9.70
*Bokobokor*	141.43** ± **15.07	35.36	23.57	28.29	57.14** ± **3.77	14.28	9.52	11.43	59.60
*Kontomire*	185.55** ± **2.27	46.39	30.93	37.11	138.76** ± **53.07	34.69	23.13	27.75	25.22
*Gboma*	220.39** ± **21.93	55.10	36.73	44.08	122.06** ± **5.19	30.52	20.34	24.41	44.61
*Alefu*	210.84** ± **71.73	52.71	35.14	42.17	68.40** ± **12.80	17.10	11.40	13.68	67.56
*Ayoyo*	67.17** ± **20.47	16.79	11.19	13.43	42.45** ± **10.69	10.61	7.08	8.49	36.79
*Lettuce*	155.64** ± **74.88	38.91	25.94	31.13	33.53** ± **9.27	8.38	5.59	6.71	78.46
*Cassava Leaves*	44.36** ± **17.37	11.09	7.39	8.87	40.57** ± **4.68	10.14	6.76	8.11	8.56

^a^RDA of 400ug/day for non-pregnant women

^b^RDA of 600ug/day for pregnant women

^c^RDA of 500ug/day for lactating women; B_9_- Folate; SD-Standard deviation. RDA-Recommended Daily Allowance

^d^Retention (Loss) values were calculated without adjustment for post-cooking mass changes and therefore represent apparent rather than true retention

## Discussion

### Intake of folate-rich vegetables among survey participants

Folate plays a critical role in DNA synthesis and foetal development, particularly in preventing neural tube defects (NTDs) and supporting maternal health during pregnancy and lactation [27]. However, dietary patterns among women in peri-urban Accra reveal significant gaps in folate intake. Data indicate low daily consumption of folate-rich vegetables like *Kontomire* (1% daily) and spinach (< 3% daily), despite their nutritional value, suggesting limited dietary diversity influenced by seasonal availability or cultural preferences [28]. A review of studies on the dietary attitudes, beliefs, and practices in Ghana revealed that fruits and vegetables are the least consumed food groups, and only about 3% of women of reproductive age consumed the recommended servings of vegetables daily, which further corroborates the low consumption reported in this study [15].The low daily intake of most vegetables aligns with broader patterns of inadequate folate consumption observed in similar populations [22, 28].

Compounding this issue, traditional cooking methods, such as prolonged boiling, may degrade folate content in leafy greens, further reducing bioavailability [28]. Socioeconomic and behavioral barriers exacerbate these challenges. While awareness of folic acid was moderate (68.3%), only 46.1% understood its role in preventing deficiencies in a previously published study among the same cohort [22]. Global comparisons underscore the need for structural interventions: in China and Nigeria, targeted prenatal education and mandatory fortification improved folate uptake, strategies underutilised in Ghana [18, 27].

To address these gaps, community-based nutrition education should emphasize periconceptional folate intake and cooking techniques that preserve nutrients [28]. Policymakers must prioritise mandatory fortification of staples like maize and wheat, subsidizing fortified products to enhance accessibility [7, 27]. Additionally, agricultural initiatives promoting underutilized folate-rich crops, such as *Alefu* and spinach, could strengthen local food systems [28].

## Moisture and folate content in GLV and the implications for reducing NTDs and OFCs

### Moisture and folate content in raw vegetables

To establish a consistent framework for discussions, the moisture content of various vegetables was evaluated. The findings indicated values ranging from 79% in cassava leaves to 93% in *Bokobokor* (water leaf), which correspond with existing literature [29, 30]. The high moisture content of these vegetables is associated with several health benefits, including improved digestion and reduced energy intake [13, 29, 30]. The high moisture content of these vegetables is associated with several health benefits, including improved digestion and reduced energy intake [13, 29, 30]. The moisture content data further explains why huge losses in folate content were observed among some of the vegetables compared to others. Folate, being a water-soluble vitamin, implies that vegetables with higher moisture content are more likely to lose more moisture, and that is the trend that was depicted in the results after 3 min of steaming over boiling water [13].

The folate content of the raw vegetable samples ranged from approximately 44 μg/100g in cassava leaves to 220 μg/100g in *Gboma*. This was consistent with previous findings in a different part of Ghana (Kumasi in the Ashanti Region), which reported total folate concentrations between 18 and 99 µg/100g for *Nephrolepis undulata* and *Kontomire* (*Xanthosoma saggitifolium),* respectively [30]. Additionally, spinach has been reported to have concentrations of 147 µg/100g [30]. Similar results (1.49 mg/kg) for raw spinach have been previously reported by other researchers [13]. However, our folate concentrations for the same samples were higher than what these studies reported. In this study, the folate content of *Gboma* was 220 µg/100g (unlike the 35 µg/100g previously reported by Wireku-Manu and colleagues [29], *Kontomire* was 185 µg/100g (unlike the 99 µg/100g [30] and spinach was 175 µg/100g (same as the 175 µg/100g reported by Czarnowska-Kujawska et al. [31] but higher than the 147 µg/100g and 149 µg/100g reported by Wireku-Manu and colleagues [30] as well as Deichier and colleagues [13] respectively). The differences in these results can be attributed to the differences in the analytical approaches, as methods lacking enzymatic deconjugation may result in lower estimates of total folate compared with methods employing enzymatic treatment as well as the soils, climatic conditions, sampling techniques, and sourcing (whether from open markets or directly from the farm) and environments within which these vegetables were grown across the different studies [11, 13, 31, 32]. Our findings highlight that consuming 100g of the fresh vegetables examined could contribute to meeting approximately 11 to 55%, 7 to 36%, and 9 to 44% of the RDA for non-pregnant, pregnant, and lactating women, respectively. As such, consuming at least 5 servings (approximately 400g) of a variety of vegetables daily, as recommended by the Ghana Food-Based Dietary Guidelines, will ensure that all women meet the daily requirements of folate [33]. Thus, local Ghanaian food resources have the potential to meet the micronutrient needs of the population if the right quantities are consumed and nutrient-saving cooking methods (which will lower folate losses in cooked vegetables) are adopted.

### Effect of cooking on folate content

The traditional cooking methods employed in Ghana stem from our food culture and foodways. They may contribute significantly to the occurrence of NTDs and OFCs as they affect the nutrient content of our cooked meals. Different cooking methods and cooking times can affect the nutrient content of foods. Some methods can help to preserve nutrients, while others can cause the loss of water-soluble vitamins and minerals or the formation of harmful compounds [13, 30, 34]. Folate is a water-soluble vitamin; as such, when the vegetables are cooked, significant losses occur through the leaching of the folates into the water for cooking [34]. Additionally, heat and ultraviolet rays have been observed to degrade folate, and as such, when vegetables are exposed to light and heat during cooking, some folates are lost [34]. In this study, the method of cooking employed was steaming, and this was done for 3 min. It is important to note that the calculation of folate loss is based on apparent retention [35]. Unfortunately, many Ghanaian dishes require extended cooking periods and frequently utilize methods such as boiling, which may last from several minutes to hours. This contributes to significant folate losses. In addition, steaming, which generally preserves more nutrients, is seldom used. Along with the lower bioavailability of natural folates in foods, these factors may partially account for the high prevalence of neural tube defects (NTDs) and orofacial clefts (OFCs) observed in Ghana [7, 9, 36]. The effects of traditional cooking methods, such as boiling and steaming, and microwave cooking have been studied, and the latter was found to be better in retention of folate than boiling [30]. The concentrations of folate that were measured in the microwaved vegetables were higher than in the raw, steamed, and boiled vegetables. This is possibly due to water loss during microwaving, which leads to more folate per unit weight of the vegetable [30].

In this research, we studied the effect of steaming on the folate content of the vegetables and found significant losses. The folate losses after 3 min of steaming ranged from approximately 9% in cassava leaves to 79% in lettuce, and this was consistent with what is reported in similar studies in Ghana and Nigeria [29, 30]. Differences in the retention or losses of micronutrients are affected by factors including vegetable type, environmental conditions, cooking technique, and the interplay between the cooking method and the specific vegetable variety [37–39]. This interplay considers disparities in the plant's structure and the exposed surface area, which are contingent on the size of the cut pieces before cooking. This implies that shorter cooking times, cutting vegetables into larger chunks, and consuming about twice as much when cooked, are advised to ensure folate retention and meet daily needs, just like other micronutrients. According to the review by Delchier and colleagues, [13] folate losses are primarily attributed to two mechanisms: (i) the leaching of folate into the surrounding liquid and (ii) oxidation during the process of heat treatment. The extent of the latter mechanism's impact varies depending on the specific vitamer under consideration. In this context, the stability of vitamers follows a descending pattern, with synthetic folic acid showing the highest stability, followed by natural folates in the order of 5 formyltetrahydrofolate (5-HCO-H4folate), 5 methyl tetrahydrofolate (5-CH3-H4folate), and ultimately, tetrahydrofolate (H4folate). It was also observed that folate losses in the understudied vegetable samples were less in those with comparatively lower moisture content, such as cassava leaves and dandelion, but greater in those with higher moisture content, such as *Kontomire*, *Gboma*, bokoborkor, lettuce, and spinach. This observation aligns with the first mechanism explained by Delchier and Colleagues, [13] supporting the leaching of the folates into the cooking water since they are water-soluble. The losses in folates after cooking, low intakes of folate-rich food resources coupled with the lower bioavailability of folates from food sources are plausible explanations as to why fruits and vegetables are abundant, especially in certain regions of the country yet marked high prevalence of folate deficiency was reported by Wegmüller et al. [18] among women of reproductive ages and similar high incidence of NTDs and OFCs among newborns in Ghana.

## Conclusion

This study demonstrates that consumption of folate-rich green leafy vegetables among adults in Accra is generally low and limited in diversity, despite the availability of locally consumed sources. Although fresh vegetables were shown to be meaningful contributors to dietary folate intake, folate losses during steaming highlight the sensitivity of this nutrient to cooking practices.

These findings underscore the importance of promoting dietary diversification and improved vegetable preparation practices as part of nutrition-sensitive public health strategies aimed at reducing folate-related adverse health outcomes. Future research should focus on comprehensive profiling of folate forms in locally available vegetables and on assessing folate bioavailability to better position these foods as complementary sources to fortified and supplemental folate.

## Supplementary Information


Supplementary Material 1.


## Data Availability

The datasets used and/or analyzed during the current study are available from the corresponding author on reasonable request.

## Abbreviations

AOAC: Association of Analytical Chemists
DGLV: Dark green leafy vegetables
NTD: Neural tube defect
LMIC: Low- and middle-income countries
HPLC: High-performance liquid chromatography
RDA: Recommended daily allowance
OFC: Orofacial cleft
FFQ: Food frequency questionnaire
FA: Folic acid
DNA: Deoxyribonucleic acid
SD: Standard deviation
SPSS: Statistical package for social sciences
HCl: Hydrochloric acid
