# Supplementary material for: Consumption patterns and quantification of folate in commonly consumed green leafy vegetables in Accra, Ghana: a cross-sectional study
Source: BMC Nutr. 2026 Jun 19;12:179. doi: 10.1186/s40795-026-01400-6 (PMC13531931; doi:10.1186/s40795-026-01400-6)
Supplement: Supplementary file 1 [file 40795_2026_1400_MOESM1_ESM.docx]

Office Use Only

Protocol Number:

NMIMR-IRB CPN: 031/21-22

**DATA COLLECTION INSTRUMENTS**

**QUESTIONNAIRE ON THE KNOWLEDGE OF FOLIC ACID DEFICIENCY AND USE OF FOLATE/ FOLIC ACID AMONG WOMEN OF CHILD-BEARING AGE**

Please provide answers to the questions below. Kindly tick where options are provided and write in the spaces provided where necessary.

Respondent ID:	Date:

**Section A: Socio-demographic Data**

1. What is your age (In completed years)
a) 15-19 years [	]
b) 20-29 years [	]
c) 30-39 years [	]
d) 40-45 years [	]
2. What is your Religion?
a) Christian
b) Muslim
c) Traditionalist
d) Other (Please specify)
3. What is your Ethnic Group?
a) Akan
b) Ewe
c) Ga-Adangbe
d) Northerner
4. What is your highest completed educational level?
a) No education [ ]
b) Primary [ ]
c) Junior High School [ ]
d) High School Education [ ]
e) High School/ vocational [ ]
f) Tertiary [ ]

5. Employment

a) Employed [ ]
b) Not employed [ ]

6. Monthly Income

a) < 599 [ ]
b) Ghȼ600-1000 [ ]
c) Ghȼ1000-1999 [ ]
d) Ghȼ2000-2999 [ ]
e) >Ghȼ3000 [ ]

7. Marital status

a) Not Married [ ]
b) Cohabiting [ ]
c) Married [ ]
d) Separated [ ]
e) Widowed [ ]

8. Gravidity (Number of times you have been pregnant)

a) 0-2
b) 3-4
c) >4

9. Parity (Number of times you have delivered a/ given birth to a child)

a) 0-2
b) 3-4
c) >4

10. Planned pregnancy

a) Yes [ ]
b) No [ ]
c) N/A

**Section B: Knowledge on Folic Acid Deficiency**

11. Folic Acid is a water-soluble vitamin

a) Yes [ ]
b) No [ ]

12. What is the best time for a woman of reproductive age to be folic acid sufficient

a) Preconception [ ]
b) After conception [ ]

13. Folic acid deficiency is of public health concern

a) Yes [ ]
b) No [ ]

14. Deficiency of folic acid may cause neural tube defects and anaemia

a) Yes [ ]
b) No [ ]

15. Which of the following foods is not a good source of naturally occurring folic acid?

a) Dark green leafy vegetable [ ]
b) Dried beans and peas (legumes) [ ]
c) Citrus fruits and juices [ ]
d) Polished rice [ ]
e) Don’t know [ ]

**Section C: Awareness and Use of Folic Acid**

16. Have you ever heard or read about folates/ Folic acid

a) Yes [ ]
b) No [ ]

Skip next question if answer to this question is No

19. What is your main source of information regarding Folic Acid

a) Physician
b) Dietitian/Nutritionist
c) Midwives/Nurse
d) TV/Radio
e) Internet

17. Do you know the importance of Folic acid?

a) Yes [ ]
b) No [ ]

18. Do you know food containing Folic acid?

a) Yes [ ]
b) No [ ]

20. Have you ever taken FA Supplements?

a) Yes [ ]
b) No [ ]

21. Are you currently taking any FA supplements?

a) Yes [ ]
b) No [ ]

**Section D: Semi-Quantitative Food Frequency Questionnaire for Folate Intake**

| **Food Item/ Food eaten** | **Frequency of Consumption** | | | | | | | **Amount per day on days eaten** | |
| --- | --- | --- | --- | --- | --- | --- | --- | --- | --- |
|  | **never** | **1/month** | **2–3/month** | **1/week** | **2–3/week** | **4–6/week** | **everyday** | **Household measures (/grams)** | **Photographs (code)** |
| **Dark green leafy vegetables** |  |  |  |  |  |  |  |  |  |
| *Kontomire* |  |  |  |  |  |  |  |  |  |
| *Ademe* |  |  |  |  |  |  |  |  |  |
| *Ayoyo* |  |  |  |  |  |  |  |  |  |
| *Gboma* |  |  |  |  |  |  |  |  |  |
| *Bokoborkor* |  |  |  |  |  |  |  |  |  |
| *Alefu* |  |  |  |  |  |  |  |  |  |
| *Lettuce* |  |  |  |  |  |  |  |  |  |
| *Spring Onion* |  |  |  |  |  |  |  |  |  |
| *Spinach* |  |  |  |  |  |  |  |  |  |
| *Dandelion* |  |  |  |  |  |  |  |  |  |
| **Legumes** |  |  |  |  |  |  |  |  |  |
| *Beans* |  |  |  |  |  |  |  |  |  |
| *Peanuts* |  |  |  |  |  |  |  |  |  |
| **Seeds and Nuts** |  |  |  |  |  |  |  |  |  |
| *Sunflower seeds* |  |  |  |  |  |  |  |  |  |
| *Sesame seeds* |  |  |  |  |  |  |  |  |  |
| *Almonds* |  |  |  |  |  |  |  |  |  |
| *Cashew nuts* |  |  |  |  |  |  |  |  |  |
| **Fruits/ Fruit Juices** |  |  |  |  |  |  |  |  |  |
| Orange |  |  |  |  |  |  |  |  |  |
| Grapefruit |  |  |  |  |  |  |  |  |  |
| Lemon/Lime |  |  |  |  |  |  |  |  |  |
| **Whole Grains** |  |  |  |  |  |  |  |  |  |
| *Oats* |  |  |  |  |  |  |  |  |  |
| *Wheat* |  |  |  |  |  |  |  |  |  |
| **Meat** |  |  |  |  |  |  |  |  |  |
| **Fish** |  |  |  |  |  |  |  |  |  |
| **Eggs** |  |  |  |  |  |  |  |  |  |
| **Seafood** |  |  |  |  |  |  |  |  |  |
| **Liver** |  |  |  |  |  |  |  |  |  |
| **Folate-fortified foods** |  |  |  |  |  |  |  |  |  |
| **FA Supplements/ Multivitamin** |  |  |  |  |  |  |  |  |  |

**Information To be Retrieved from Hospital Records**

1. Name of Health Facility
2. Region
3. Number of NTDs cases
4. Age in completed years/months
5. Gender
   a. Male
   b. Female
6. Clinical diagnosis
7. Type of anomaly
   a. Anencephaly
   b. Hydrocephaly
   c. Cleft Lip
   d. Cleft palate
   e. Cleft Lip and palate
   f. Spina Bifida
8. Management outcomes.
